# Statistical Disease Progression Modeling in Alzheimer Disease

**DOI:** 10.3389/fdata.2020.00024

**Published:** 2020-08-12

**Authors:** Lars Lau Raket

**Affiliations:** ^1^H. Lundbeck A/S, Copenhagen, Denmark; ^2^Clinical Memory Research Unit, Department of Clinical Sciences, Lund University, Lund, Sweden

**Keywords:** cognitive decline, dementia, Alzheimer disease, disease staging, biomarkers, disease progression modeling, progression curves, cognitive reserve

## Abstract

**Background:** The characterizing symptom of Alzheimer disease (AD) is cognitive deterioration. While much recent work has focused on defining AD as a biological construct, most patients are still diagnosed, staged, and treated based on their cognitive symptoms. But the cognitive capability of a patient at any time throughout this deterioration reflects not only the disease state, but also the effect of the cognitive decline on the patient's pre-disease cognitive capability. Patients with high pre-disease cognitive capabilities tend to score better on cognitive tests that are sensitive early in disease relative to patients with low pre-disease cognitive capabilities at a similar disease stage. Thus, a single assessment with a cognitive test is often not adequate for determining the stage of an AD patient. Repeated evaluation of patients' cognition over time may improve the ability to stage AD patients, and such longitudinal assessments in combinations with biomarker assessments can help elucidate the time dynamics of biomarkers. In turn, this can potentially lead to identification of markers that are predictive of disease stage and future cognitive decline, possibly before any cognitive deficit is measurable.

**Methods and Findings:** This article presents a class of statistical disease progression models and applies them to longitudinal cognitive scores. These non-linear mixed-effects disease progression models explicitly model disease stage, baseline cognition, and the patients' individual changes in cognitive ability as latent variables. Maximum-likelihood estimation in these models induces a data-driven criterion for separating disease progression and baseline cognition. Applied to data from the Alzheimer's Disease Neuroimaging Initiative, the model estimated a timeline of cognitive decline that spans ~15 years from the earliest subjective cognitive deficits to severe AD dementia. Subsequent analyses demonstrated how direct modeling of latent factors that modify the observed data patterns provides a scaffold for understanding disease progression, biomarkers, and treatment effects along the continuous time progression of disease.

**Conclusions:** The presented framework enables direct interpretations of factors that modify cognitive decline. The results give new insights to the value of biomarkers for staging patients and suggest alternative explanations for previous findings related to accelerated cognitive decline among highly educated patients and patients on symptomatic treatments.

## Background

Alzheimer disease (AD) is slowly progressing with preclinical and prodromal phases lasting many years before the onset of dementia. The stage of the underlying disease process of an AD patient entering a clinical trial is largely unknown, but may be estimated by a combination of, for example, cognitive testing, clinical evaluation, and biomarker results. While these procedures for evaluating disease severity are useful for creating coarse groupings of patients, the factors used to create groupings may be systematically affected by a wealth of factors not directly tied to the disease process, for example, comorbidities, intelligence, level of education, and genetics.

So far, efforts to develop therapies that delay or halt the progression of AD have generally been unsuccessful, and the vast majority of trials testing symptomatic agents in AD have failed. These failures may be due to wrong therapeutic targets or non-efficacious therapies, but it is conceivable that a proportion of trial failures could be attributed to other factors, such as study design, endpoints, and non-optimal patient population selection. For disease-modifying drugs, for example, the current standard durations for interventional studies may not be adequate. Simulations based on cohort studies suggest that prevention of disease in cognitively normal individuals may require study lengths far beyond the current standard to achieve high statistical power for detecting an effect of even very efficacious drugs (Anderson et al., 2017; Insel et al., 2019). Better patient selection and an improved understanding of patient-level cognitive decline could potentially address this problem.

### Cognitive Decline and Symptom Onset

The characterizing symptom of AD is cognitive deterioration. The cognitive capability of a patient at any time throughout this deterioration will not directly reflect the disease state, but the cumulative effect of the cognitive decline on the patient's pre-disease cognitive capability.

Many factors influence instantaneous cognitive ability, and low cognitive ability at a single time point is not necessarily an indication of cognitive decline. Cognitive decline can only be established by repeated evaluations of patients' cognition over time. Longitudinal assessments of patient cognition also offer the benefit of hindsight—once cognitive decline or dementia is established, one can traverse back in time along the cognitive trajectory and predict when the decline started and search for patterns that are indicative of future cognitive decline in its earliest stages. If done properly, one can synchronize individual observed trajectories to one long-term timeline representative of the full span and variation of cognitive decline over the course of disease.

### Disease Progression Modeling

Alzheimer disease typically presents in a sporadic late-onset form. The autosomal dominant forms of AD (ADAD) caused by rare genetic mutations have earlier onset than sporadic AD, but otherwise, the pathogenesis is largely similar (Bateman et al., 2011). In ADAD, age at symptom onset is strongly affected by mutation type, parental age at symptom onset, APOE genotype, and sex (Ryman et al., 2014). These factors can be used to calculate expected patient age at symptom onset for ADAD patients, which can be used to construct a more synchronized time scale for studying biomarkers and the pathological cascade of the disease (Wang et al., 2019). Furthermore, this makes it possible to do primary prevention studies in a highly efficient manner (Bateman et al., 2017).

In sporadic AD, age at onset cannot be predicted accurately from demographic or genetic factors. Assessment of biomarkers, such as amyloid and tau load in cerebrospinal fluid (CSF) or by positron emission tomography (PET) may be used to diagnose the disease even in the earliest stages (Jack et al., 2018), but such assessments can be both invasive and expensive, and data are sparse. There are, however, rich datasets with longitudinal cognitive measurements that span different parts of the disease. An appealing use of this data is to assemble the individual observed short-term trajectories to one long-term timeline representative of the full span of cognitive decline over the disease.

Different approaches to construct disease progression models for AD have been taken. A classic approach is to formulate the changes in cognitive scores using differential equations (Ito et al., 2011; Gomeni et al., 2012; Samtani et al., 2012; Delor et al., 2013). One major drawback of this type of modeling is that covariate effects and different sources of random variation should be formulated in the differential equation framework and may be very difficult to handle and interpret. A more direct approach to disease progression modeling in AD is event-based models (Young et al., 2014; Oxtoby et al., 2018) where cutoff points of abnormality are inferred from observed biomarkers or clinical scales, and disease stage is mapped to a discrete set of biomarker-abnormality events. Event-based models can improve robustness, but the dichotomization of variables also reduces the granularity of the results, especially for variables that do not show a bimodal distribution and/or continuously evolve with disease progression.

An alternative class of disease progression models relies on direct modeling of the observed longitudinal trajectories and explicit modeling of the patient-level disease stage (Jedynak et al., 2012). An important example of this type of approach is the model by Donohue et al. (2014), which simultaneously models multiple observations of cognitive measures and biomarkers. This modeling approach has been powerful in illuminating the multivariate nature of AD progression. The approach was recently generalized to a wider class of Bayesian latent-time joint mixed-effects models (Li et al., 2018). This generalized class of models allows dependencies between different outcomes and inclusion of covariates, but covariates can only model variation in outcomes and not disease stage or progression rate.

For modeling disease progression of very high-dimensional data with rich structure, such as brain imaging, disease progression models are often considered in the context of Riemannian geometry (Louis et al., 2019). While there have been recent advances in the range implementable models (Schiratti et al., 2017; Koval et al., 2018), the complexity and computational demand are still restricting the types data and effects that can be modeled by these approaches.

For practical applications of disease progression modeling, there are several important considerations to make. Simultaneous modeling of multiple outcomes is desirable as one can detect signals in multiple outcomes that may reduce noise in the staging of patients. However, it typically comes with an assumption of all outcomes being synchronized in the same disease time model (Donohue et al., 2014; Li et al., 2018). Therefore, care should be taken when deciding which outcomes to include. For example, if a group of individuals with different age-related neurodegenerative diseases is modeled, these individuals may all experience progressive dementia that can be mapped to a common trajectory of cognitive decline, but their individual biomarker measurements may be very different along this trajectory, and including these as outcomes may deteriorate the quality of the staging. Conversely, if most individuals have the same cause of their cognitive decline (e.g., AD), including biomarkers may help staging patients in the early stages of disease where no or little cognitive deficit is detectable. Another important consideration is the time scale at which to model disease progression. Age is typically considered the major risk factor for developing AD, but age at first diagnosis of AD can vary by decades between patients, and because this span is much greater than the entire course of cognitive decline associated with AD, patient age is not an appropriate scale for understanding the pattern of cognitive decline in AD because it may amplify the *a priori* dis-synchronization between patients by orders of magnitude. For example, two individuals diagnosed with AD dementia at 60 and 90 years of age, respectively, may have similar courses of cognitive decline, but an age-indexed model would have to compensate for the additional 30 years' difference when compared to a diagnosis-indexed model. The negative consequence of this can, for example, be seen in Figure 1 in Li et al. (2018), where patient-level trajectories go from minimal to maximal severity over 10–15 years, while variation of when maximal severity is reached between patients is spread out over 30-years periods. Therefore, a more natural scale for studying the patterns of cognitive decline is time since symptom onset. However, self- or caregiver-reported age at symptom onset is not perfect either. It may be imprecise because of the patient's memory problems; recall bias, where early sporadic cognitive issues are believed to be symptoms of the disease; or personal differences in sensitivity and interpretation of the earliest cognitive problems.

In this article, we propose a new approach to disease progression modeling that separates disease stage and deviations from the mean pattern in a fully data-driven manner. The model enables more detailed modeling and analysis of some of the aspects of cognitive decline compared to previous models. For example, it allows investigation of whether observed variables are related to cognitive ability, disease stage, or rate of decline. In the presented form, the model is estimating a disease timeline from repeated assessments of a univariate measure, such as a cognitive scale. The model is inspired by the statistical framework presented by Raket et al. (2014), where systematic patterns of variation in both vertical (observed cognitive score) and horizontal (disease timing) directions are modeled simultaneously on both the population and individual levels. The model allows covariate effects on both outcomes and disease progression, and all model parameters are estimated simultaneously using maximum likelihood estimation.

The goal of this work was to explore whether the proposed disease progression model could align observed cognitive trajectories to a precise timeline of cognitive decline associated with AD and to evaluate if this modeling would shed new light on aspects related to disease progression and biomarkers. When the model was fitted to cognitive scores from Alzheimer's Disease Neuroimaging Initiative (ADNI), the presented model aligned the cognitive trajectories of patients to a consistent shape of cognitive decline with a span of ~15 years from the earliest subjective cognitive deficits to severe AD dementia. It was shown that the model's predictions of patients' disease stages based on their longitudinal cognitive scores could predict time since symptom onset and diagnosis. It was further demonstrated that the predicted disease stages provided a more suitable time scale for modeling the evolution of biomarkers over the course of disease than group-wise modeling based on patient symptoms at baseline. The model was used to estimate the effects of sex, age, and education on cognitive decline and to evaluate the effects of cholinesterase inhibitor (ChEI) treatment on cognitive decline. Finally, the model was fitted to the cognitive trajectories of a subset of patients with a rich set of biomarkers available at baseline to estimate if baseline biomarker profile could predict disease stage. The results of the model in an independent held-out validation dataset confirmed that baseline biomarker profiles could predict the disease stage of unseen individuals—even in the preclinical phases of disease where no clinically detectable cognitive impairment was present.

## Methods

### Data

Data used in the preparation of this article were obtained from the ADNI database (adni.loni.usc.edu). The ADNI was launched in 2003 as a public–private partnership, led by the principal investigator Michael W. Weiner, MD. The primary goal of ADNI has been to test whether serial magnetic resonance imaging (MRI), PET, other biological markers, and clinical and neuropsychological assessment can be combined to measure the progression of mild cognitive impairment (MCI) and early AD. For up-to-date information, see www.adni-info.org.

Patients included in the current study were required to have a valid classification at baseline [cognitively normal, significant memory concern, MCI (early), MCI (late), or dementia].

#### Outcomes

The main outcome measure considered was the total score of the 13-item Alzheimer's Disease Assessment Scale–Cognitive Subscale (ADAS-Cog; range = 0–85; lower score indicates less impairment) (Mohs et al., 1997). Included patients were required to have at least one valid ADAS-Cog total score to be included in the present study.

Other outcomes reported were onset of various symptoms related to cognitive impairment and AD; Clinical Dementia Rating scale—sum of boxes (Hughes et al., 1982); Functional Activities Questionnaire (Pfeffer et al., 1982); fluorodeoxyglucose (FDG) PET meta region of interest (meta-ROI) (Landau et al., 2011); cross-sectional hippocampal volume extracted from MRI using FreeSurfer (Fischl, 2012); florbetapir PET SUVr (Landau et al., 2015); Aβ_1−42_, total tau, and *p*-tau_181_ concentrations in CSF as measured using the Roche Elecsys^®^ immunoassay (Bittner et al., 2016); the ratio of Aβ_1−42_/Aβ_1−40_ concentrations in CSF as measured by two-dimensional ultraperformance liquid chromatography tandem mass spectrometry; and neurofilament light chain (NfL) levels in plasma measured using a single-molecule array platform (Mattsson et al., 2019).

### Disease Progression Model

Let *y*_*ij*_ represent the observed cognitive score of patient *i* at time *t*_*ij*_ (*i* = 1, …, *n*, *j* = 1, …, *m*_*i*_). We assume that *y*_*ij*_ is generated by a model of the form

$$\begin{array}{l} y_{ij}=\theta \left(w_{i}\left(t_{ij}\right)\right)+x_{i}\left(t_{ij}\right)+\varepsilon_{ij} \end{array}$$

where θ is a function that represents the shape of cognitive decline; *w*_*i*_ is a *warping* function that transforms observation time *t*_*ij*_ to a disease time scale *w*_*i*_(*t*_*ij*_) that is aligned across patients; *x*_*i*_ is the idiosyncratic patient-level deviation from the mean shape that represents consistent deviations over time; and ε_*ij*_ is independent measurement noise.

Cognitive scores can be extremely noisy because of many different sources of variation, and one will have to make suitable model choices to accurately infer the shape of the disease timeline of cognitive decline θ, to predict patient-level disease stage *w*_*i*_, and to predict the entire patient-level course of decline ŷ_*i*_. In the following, we describe the basic model choices taken here and their motivations.

Because we are modeling cognitive decline in pathological aging, it is natural to assume that the representative shape of decline θ is a function that has a stable left asymptote (pre-disease cognitive normality) and a monotone decline. In this article, we focus on ADAS-Cog scores that show a distinct exponential decline in dementia (Yang et al., 2011), and thus we will work with a parametrized family of exponential functions to model the mean progression pattern

$$\begin{array}{l} \theta \left(t\right)=l\cdot \exp\left(\frac{t+s}{\exp\left(g\right)}\right)+v, \end{array}$$

It is worth noting that this choice of θ is overparametrized unless restrictions are put on some of the parameters. The constraint used here (discussed further below) is that *s* = 0 for the cognitively normal individuals, in which case *v* is the left asymptote representing the average stable pre-disease cognitive score and where the remaining parameters determine the shape of the decline.

The mean progression pattern θ can be modeled differently to achieve other properties, for example, as a generalized logistic function or as a monotone spline (Ramsay, 1988). Other modeling options are available in the progmod R package (Raket, 2020) accompanying this article.

The mapping of observed time to disease time *w*_*i*_ should allow the model to assemble short-term longitudinal observations to a long-term timeline of cognitive decline. Because the major source of horizontal variation can likely be ascribed to differences in how long the patient has had the disease before we begin observing them, we model *w*_*i*_ as a shift of study time.

$$\begin{array}{l} w_{i}\left(t\right)=t+s_{i}. \end{array}$$

#### Random Effects

When modeling longitudinal data for groups of individuals, it is often natural to describe systematic differences between individuals using random effect. The proposed disease progression model has three types of random effects.

- *s*_*i*_: Random patient-level shift that models the disease stage of patient *i*. Assumed to follow a zero-mean normal distribution with unknown variance τ^2^.
- *x*_*i*_: Random patient-level systematic deviation from the mean curve. Assumed to be a sum of discrete-time observation of a Brownian motion *x*_*i, BM*_ and an independent zero-mean normally distributed starting level *x*_*i*, 0_ with unknown variance. The covariance function of *x*_*i*_ is thus C(*t, t*′) = $\sigma_{\text{BM}}^{2}\;\cdot \text{ min}\left(t,t^{\prime }\right)\;+\;\sigma {\;}_{0}^{2}$ where $\;\sigma {\;}_{BM}^{2}$ is an unknown parameter controlling variance scale of the Brownian motion, and $\;\sigma {\;}_{0}^{2}$ is an unknown parameter controlling the variance of the random starting level.
- ε_*ij*_: Random observation noise. Assumed to be independent zero-mean normally distributed with unknown variance σ^2^.

A free correlation between *s*_*i*_ and the starting level *x*_*i*, 0_ is included in the model; the remaining effects are assumed independent.

#### Fixed Effects

The basic model parameters *l*, *g*, *s*, and *v* that describe the shape of θ are modeled as fixed effects.

- *l* is a scaling parameter of the exponential function. Because a goal of disease progression modeling is to find a common pattern of decline, *l* will be modeled as a single free parameter.
- *g* is a scaling parameter of time. Patient-level differences in rate of decline that can be ascribed to a covariate or factor can be modeled as a regression-type model on *g*. Initially, this parameter will be modeled as a single free parameter.
- *s* is a shift of observed time. Patient-level differences in disease stage that can be ascribed to a covariate or factor can be modeled as fixed effects. Because the present study includes several cohorts at different disease stages (e.g., cognitively normal individuals, patients with dementia), the initial modeling will have different *s* parameters for non-cognitively normal cohorts and *s* = 0 for the cognitively normal individuals to ensure structural identifiability of the model (Lavielle and Aarons, 2016). Thus, *s* is modeling disease time since the average baseline stage of the cognitively normal individuals.
- *v* is an intercept parameter describing the left asymptote. Patient-level differences in pre-disease cognition that can be ascribed to a covariate or factor can be modeled as a regression-type model on *v*. Initially, this parameter will be modeled as a single free parameter.

#### Final Model Formulation

The basic model used has the form

$$\begin{array}{l} y_{ij}=l\cdot \exp\left(\frac{t_{ij}+s_{i}+X_{s,ij}\beta_{s}}{\exp\left(g\right)}\right)+v+x_{i,\;BM}\left(t_{ij}\right)+x_{i,\;0}+\varepsilon_{ij} \end{array}$$

where *l, g, v* ϵ R and $\beta_{s}\in R^{4}$ are free fixed effects, and *X*_*s, ij*_ is the four-dimensional dummy row-vector indicating which, if any, of the symptomatic baseline groups individual *i* belongs to. The random effects follow a joint zero-mean normal distribution

$$\left(\begin{array}{c} \begin{array}{c} s_{i}\\ x_{i,\;0} \end{array}\\ x_{i,\;BM}\left(t_{ij}\right)+x_{i,\;0}+\varepsilon_{ij}\\ x_{i,\;BM}\left(t_{ik}\right)+x_{i,\;0}+\varepsilon_{ik} \end{array}\right)\sim N\left(\left(\begin{array}{c} \begin{array}{c} 0\\ 0 \end{array}\\ 0\\ 0 \end{array}\right),\left[\begin{array}{ccc} \begin{array}{cc} \tau^{2} & \rho \\ \rho & \sigma_{0}^{2} \end{array} & \begin{array}{c} \rho \\ \sigma_{0}^{2} \end{array} & \begin{array}{c} \rho \\ \sigma_{0}^{2} \end{array}\\ \begin{array}{cc} \rho & \sigma_{0}^{2} \end{array} & \sigma_{BM}^{2}\cdot t_{ij}+\sigma_{0}^{2}+\sigma^{2} & \sigma_{BM}^{2}\cdot \min\left(t_{ij},t_{ik}\right)+\sigma_{0}^{2}\\ \begin{array}{cc} \rho & \sigma_{0}^{2} \end{array} & \sigma_{BM}^{2}\cdot \min\left(t_{ij},t_{ik}\right)+\sigma_{0}^{2} & \sigma_{BM}^{2}\cdot t_{ik}+\sigma_{0}^{2}+\sigma^{2} \end{array}\;\right]\right)$$

where τ^2^, $\sigma_{BM}^{2}$, $\sigma_{0}^{2},\;\sigma^{2}>0$ are unknown variance parameters, and ρ∈*R* is a parameter that controls the correlation between the random time shift *s*_*i*_ and the random starting level *x*_*i*, 0_.

### Statistical Analysis

Estimation in the disease progression model was done with maximum likelihood using the two-step algorithm of Lindstrom and Bates (1990). Random effects were predicted as the most likely values given the data and maximum likelihood parameter estimates (i.e., they maximized the posterior under the parameter estimates).

To investigate the effect of covariates on the pattern of disease progression, forward selection was used to evaluate models with all combinations of covariate effects on rate of decline *g*, disease stage *s*, and pre-disease cognition *v*. The search was continued as long as the Akaike Information Criterion (Akaike, 1998) improved, but the model selection was based on the more conservative Schwarz's Bayesian Information Criterion (BIC) (Schwarz, 1978).

To investigate if predicted disease time was predictive of time since reported symptom onset, linear regression was done on time since reported symptom onset (at baseline) using predicted disease time as a covariate. *P*-values were computed using *t*-tests.

Linear mixed-effects modeling was used to investigate if predicted disease time offered a better time scale for modeling other longitudinal outcomes (e.g., biomarkers) than time since baseline for the five baseline groups. To allow for non-linear trends in the mean pattern, the outcome was modeled using a cubic B-spline function with 3 degrees of freedom plus an intercept across predicted disease time and time since baseline (one pattern per baseline group), respectively. Patient-level random slopes and intercepts were included to model longitudinal deviations within an individual. *P*-values were computed using likelihood ratio tests with maximum likelihood estimation.

Comparisons of quantitative outcomes between groups with two levels were done using Wilcoxon rank sum tests, and correlations were evaluated with Spearman rank correlation coefficients.

#### Software

All analyses were done using R version 4.0.0 (R Core Team, 2020). Maximum likelihood estimation in the disease progression models was done using the progmod R package (Raket, 2020), which builds on the estimation procedures available in the nlme and covBM R packages (Pinheiro et al., 2019).

## Results

### Basic Model

**Figure 1:**
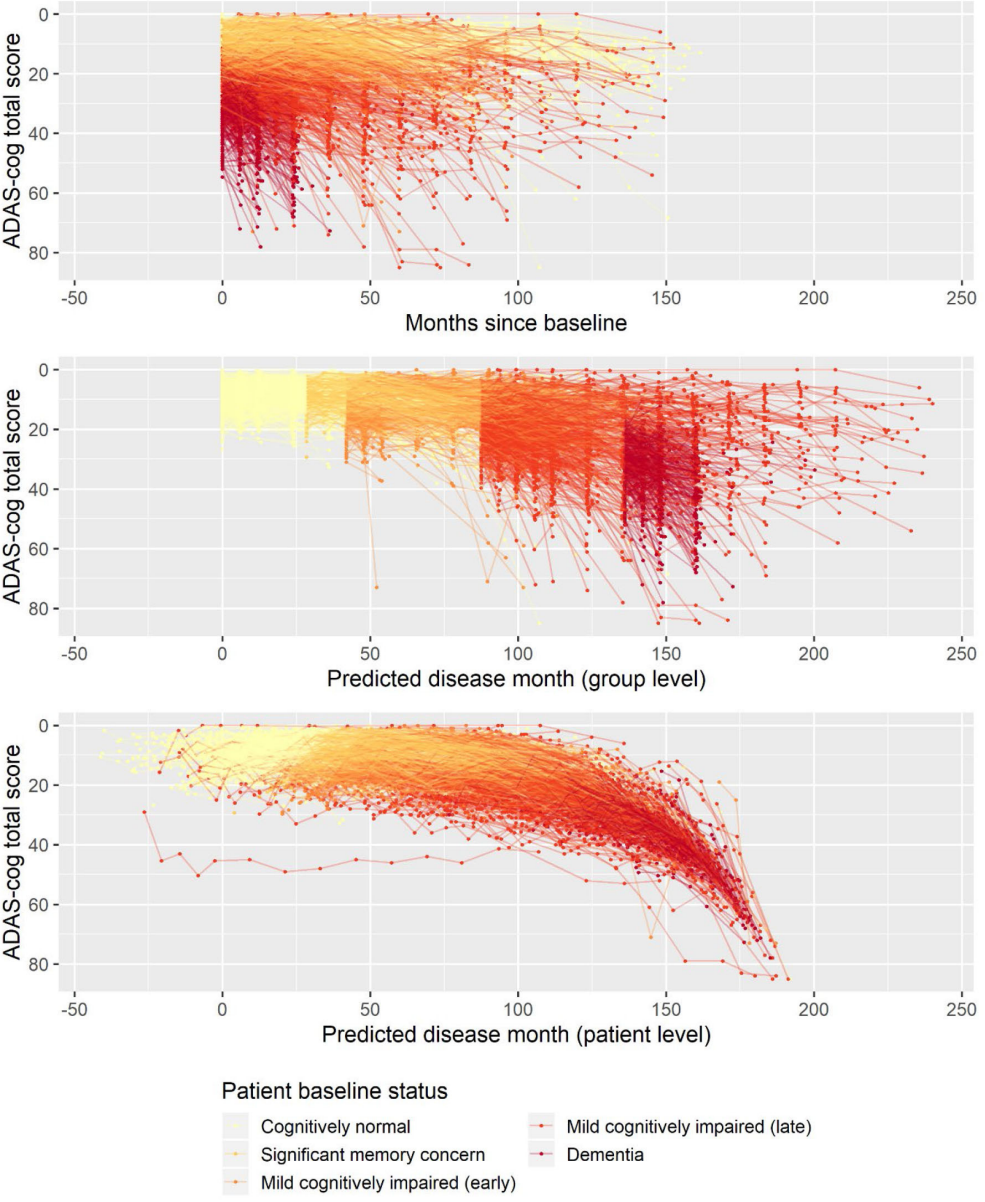
Observed longitudinal ADAS-Cog trajectories for 2,142 ADNI participants plotted against time in study (top), predicted disease time based on the fixed-effects staging of the different patient baseline status groups relative to the cognitively normal group (middle), and predicted individual disease time based on both fixed group and random individual effects.

The basic model described above was fitted on longitudinal ADAS-Cog data from ADNI. The data comprised 9,830 ADAS-Cog scores across 2,142 individuals. The ADAS-Cog scores plotted against study time are shown in the top panel of Figure 1. The middle panel in Figure 1 shows the fixed-effects staging of the baseline status groups relative to the cognitively normal group on the predicted time scale (“disease month”). The bottom panel of Figure 1 shows the predicted individual staging (both fixed and random effects) of trajectories on the predicted time scale. Relative to the average baseline disease stage of the cognitively normal group, the model estimated that the significant memory concern group was 29 months later into the trajectory of cognitive decline, whereas the early and late MCI groups were, respectively, 42 and 88 months later and that the dementia group was 136 months later. The model had 12 degrees of freedom, and twice the negative log likelihood of the fitted model was 59,468.52. AIC and BIC were 59,492.52 and 59,578.84, respectively.

### Validation of the Basic Model

The presented disease progression model aggregates the information in baseline status groups and the longitudinal trajectories of participants to a single number, the predicted disease month. For this continuous disease progression scale to be relevant to AD, it should also hold information that describes other aspects of the disease than the cognitive deterioration observed on ADAS-Cog that the model was fitted on.

**Figure 2:**
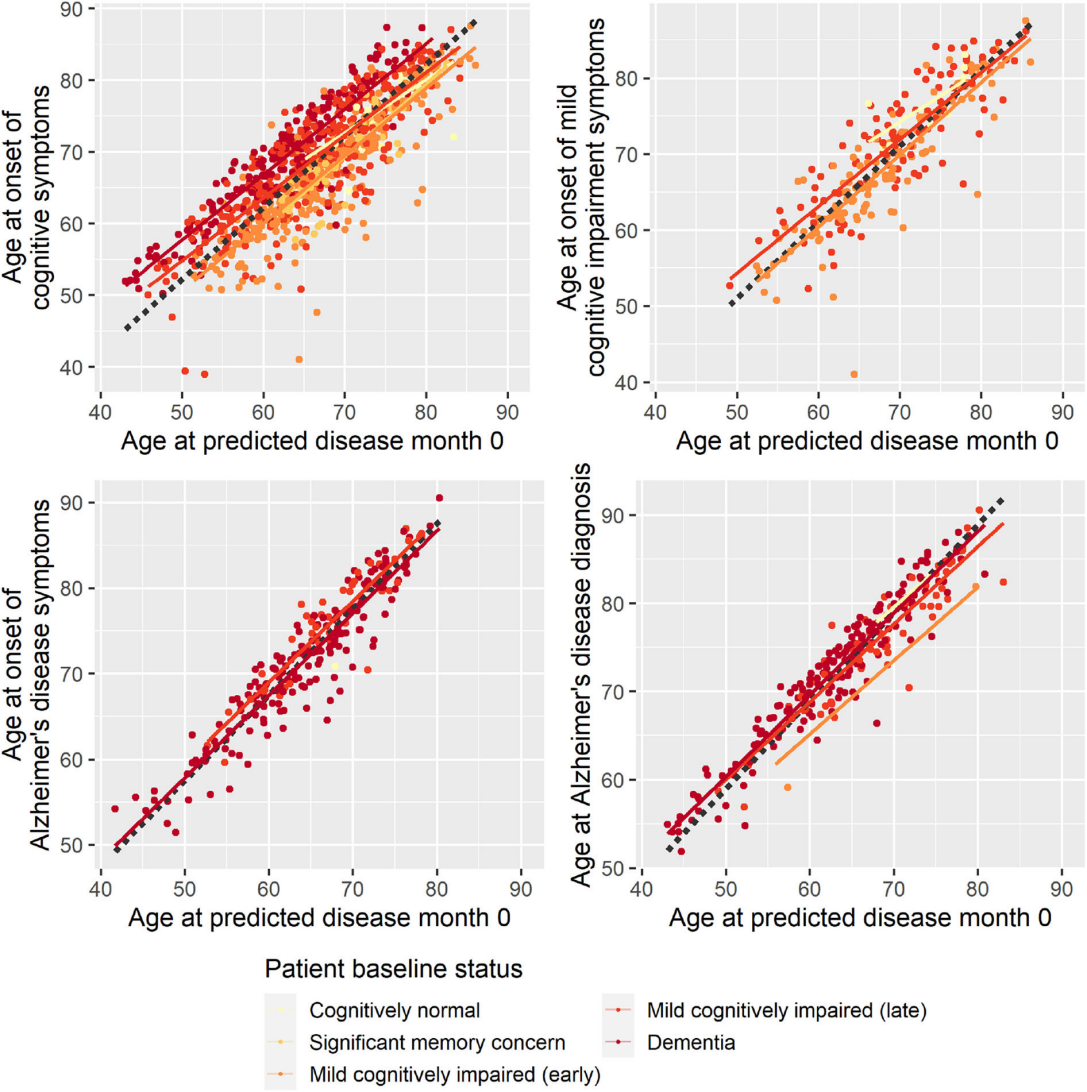
Reported age at onset of cognitive symptoms (top left), cognitive impairment symptoms (top right), Alzheimer disease symptoms (bottom left), and Alzheimer disease diagnosis (bottom right) as a function of age at predicted disease month 0. Dotted lines represent the best-fitting least-squares estimated lines with slope 1.

To evaluate whether the disease progression model captured milestones of cognitive deterioration, we investigated the model's ability to predict self-reported onset of cognitive symptoms, MCI symptoms, AD symptoms, or diagnosis of AD. There were 1,142 participants who had at least one entry of these data during the study follow-up. Age at symptom onset or diagnosis plotted against the age at the model's predicted disease time 0 (computed as age at baseline minus predicted shift in disease time in years) is shown in Figure 2. In an ideal setting where trajectories were perfectly aligned and onset/diagnosis would be perfectly consistently reported across individuals, the results of each measure would lie on a line with slope 1, and the intercept would represent the difference in years between age at disease time 0 and the age at onset/diagnosis time. For the age at onset of cognitive symptoms, there seem to be different intercepts for the different baseline groups, where more severe baseline groups tend to report symptom onset later relative to the model prediction of the less severe groups. This may be an effect of different subjective definitions of onset of cognitive symptoms across baseline groups, it may be because of biased model estimates of the staging of the baseline groups, or a combination.

Based on linear regression, predicted disease month was predictive of time since cognitive symptom onset (*p* < 0.0001), time since AD symptoms onset (*p* < 0.0001), and time since Alzheimer diagnosis (*p* < 0.0001)—all times relative to study baseline. Predicted disease month was not significantly predictive for time since MCI symptom onset (*p* = 0.558).

**Figure 3:**
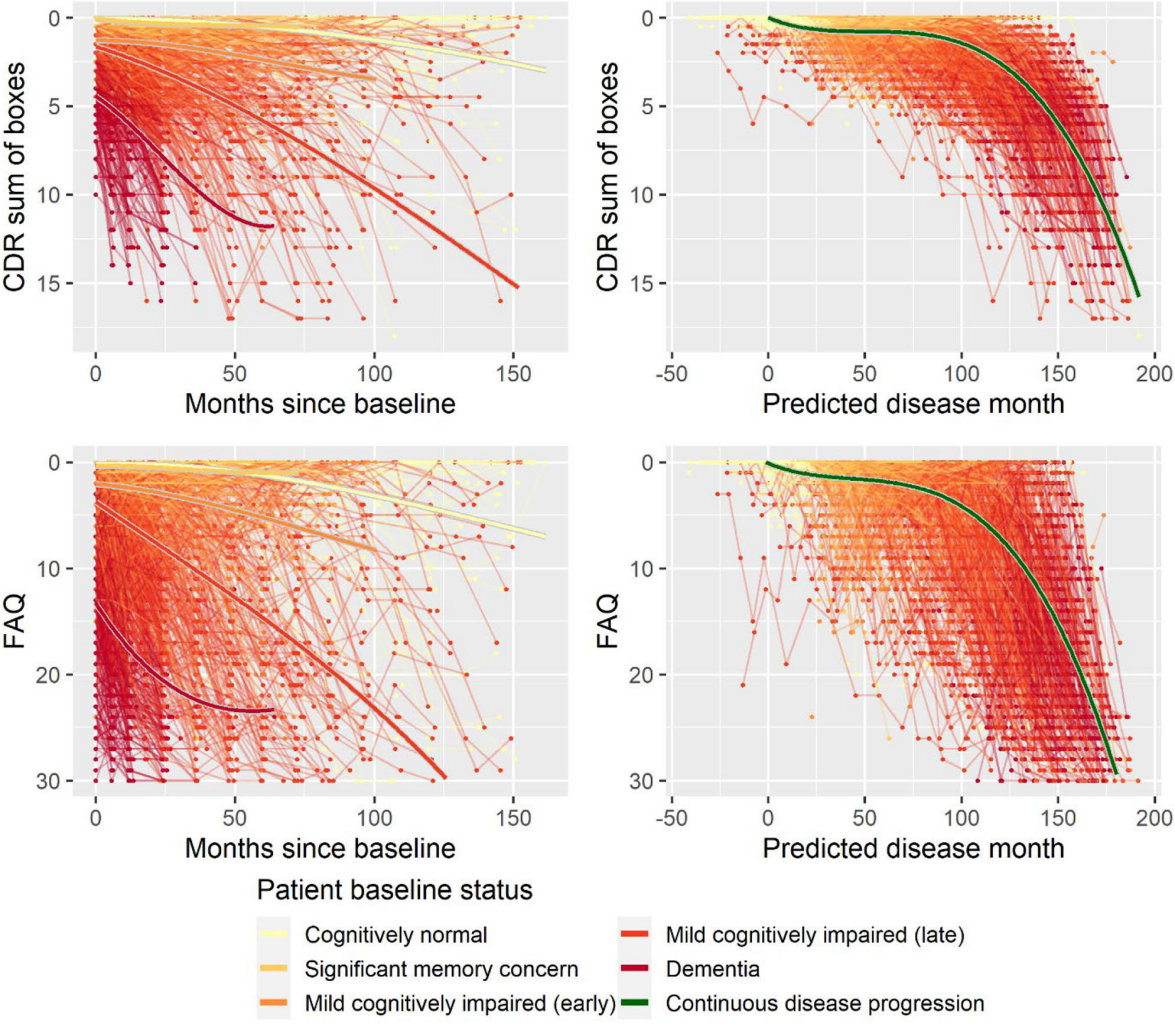
Data and estimated biomarker trajectories for CDR sum of boxes and FAQ. Left column shows results when allowing different trajectories for the five baseline groups; right column shows the results when requiring a single trajectory over predicted disease time.

**Figure 4:**
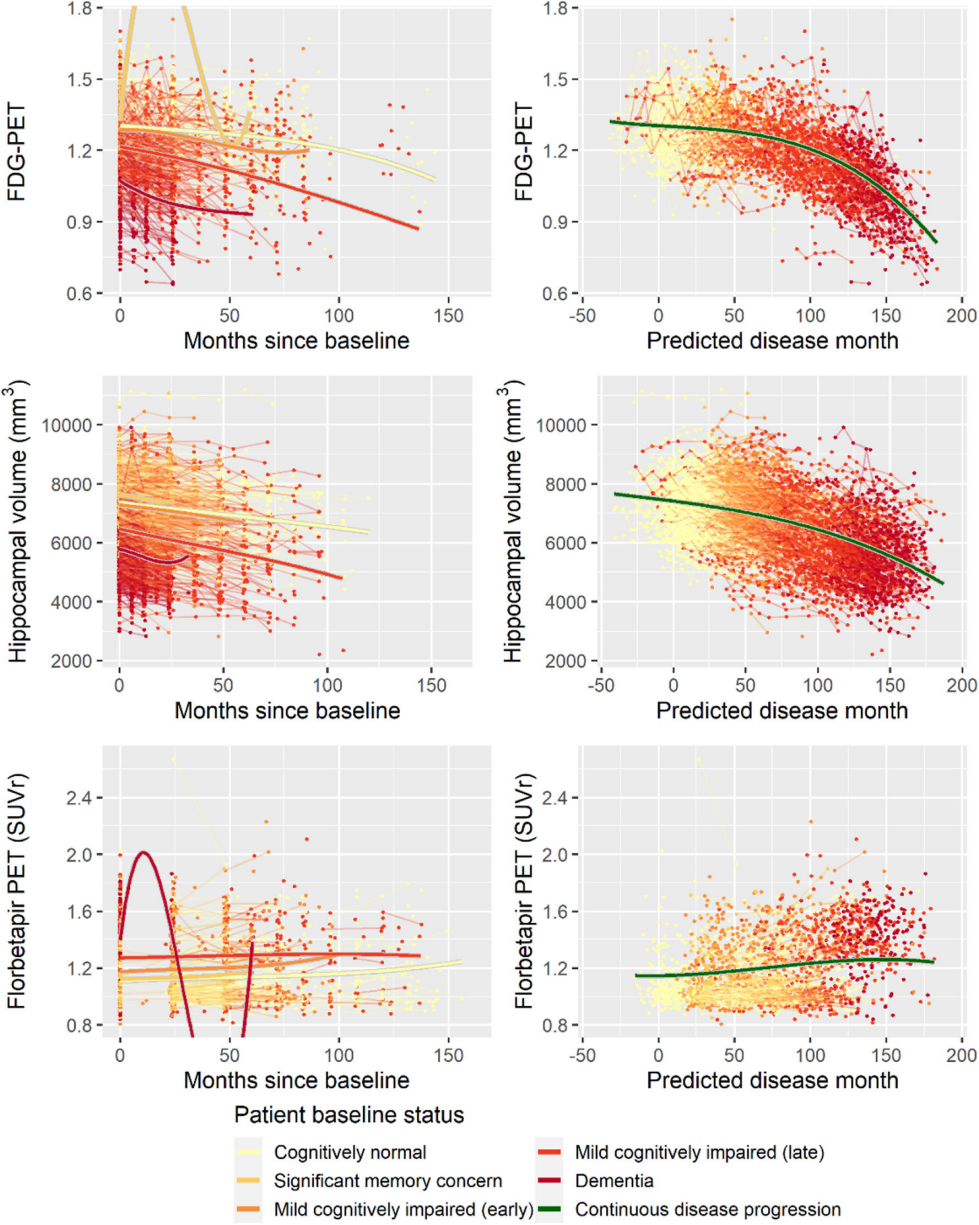
Data and estimated biomarker trajectories for FDG-PET, hippocampal volume (MRI), and florbetapir PET. Left column shows results when allowing different trajectories for the five baseline groups; right column shows the results when requiring a single trajectory over predicted disease time. Note that the oscillations for the significant memory concern and dementia groups on FDG-PET and florbetapir PET, respectively, occur in time periods where no data were collected for these groups.

**Figure 5:**
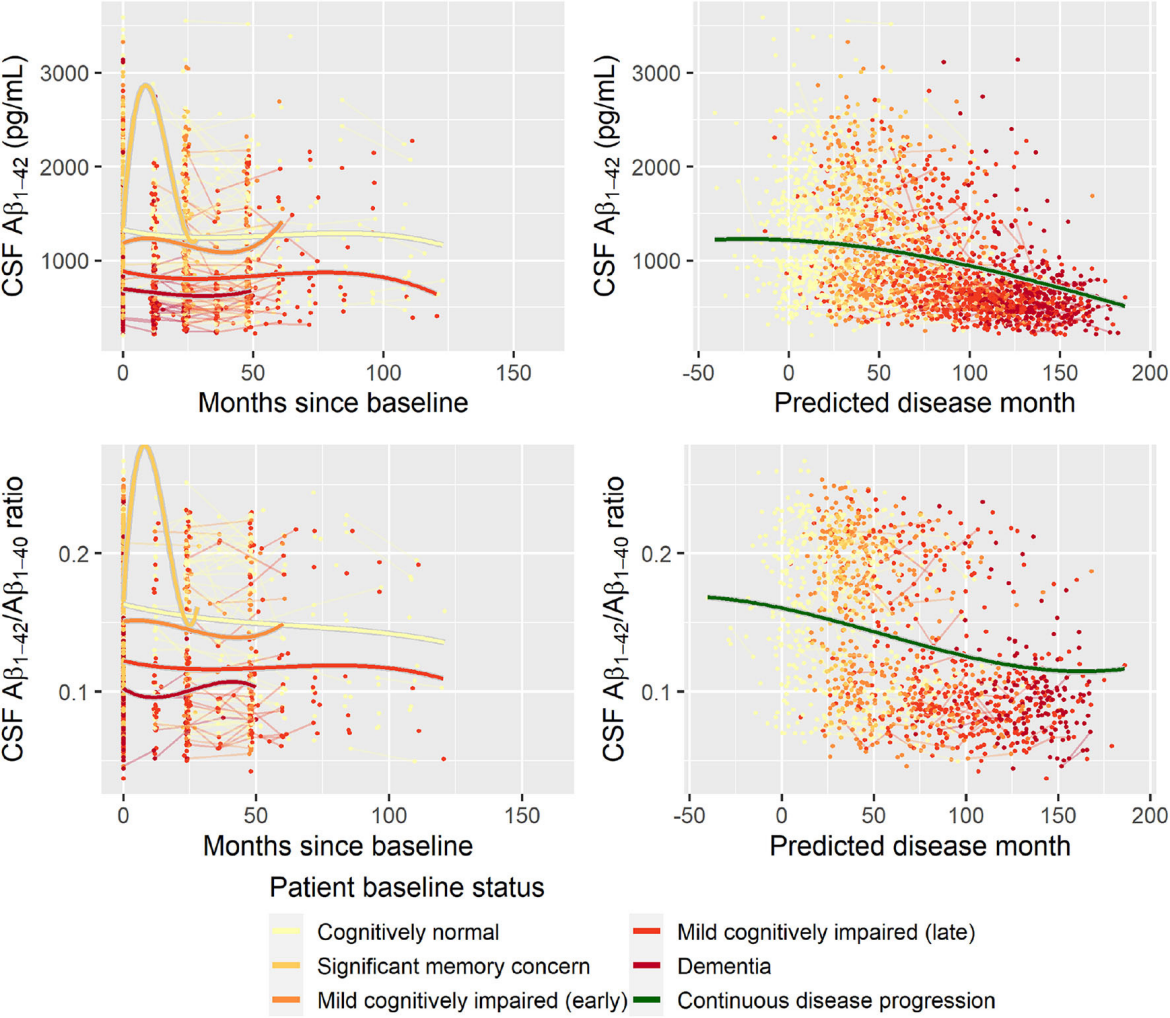
Data and estimated biomarker trajectories for Aβ_1−42_ (CSF) and Aβ_1−42_/Aβ_1−40_ ratio (CSF). Left column shows results when allowing different trajectories for the five baseline groups; right column shows the results when requiring a single trajectory over predicted disease time. Note that the oscillations for the significant memory concern group occur in time periods where no data were collected for this group.

**Figure 6:**
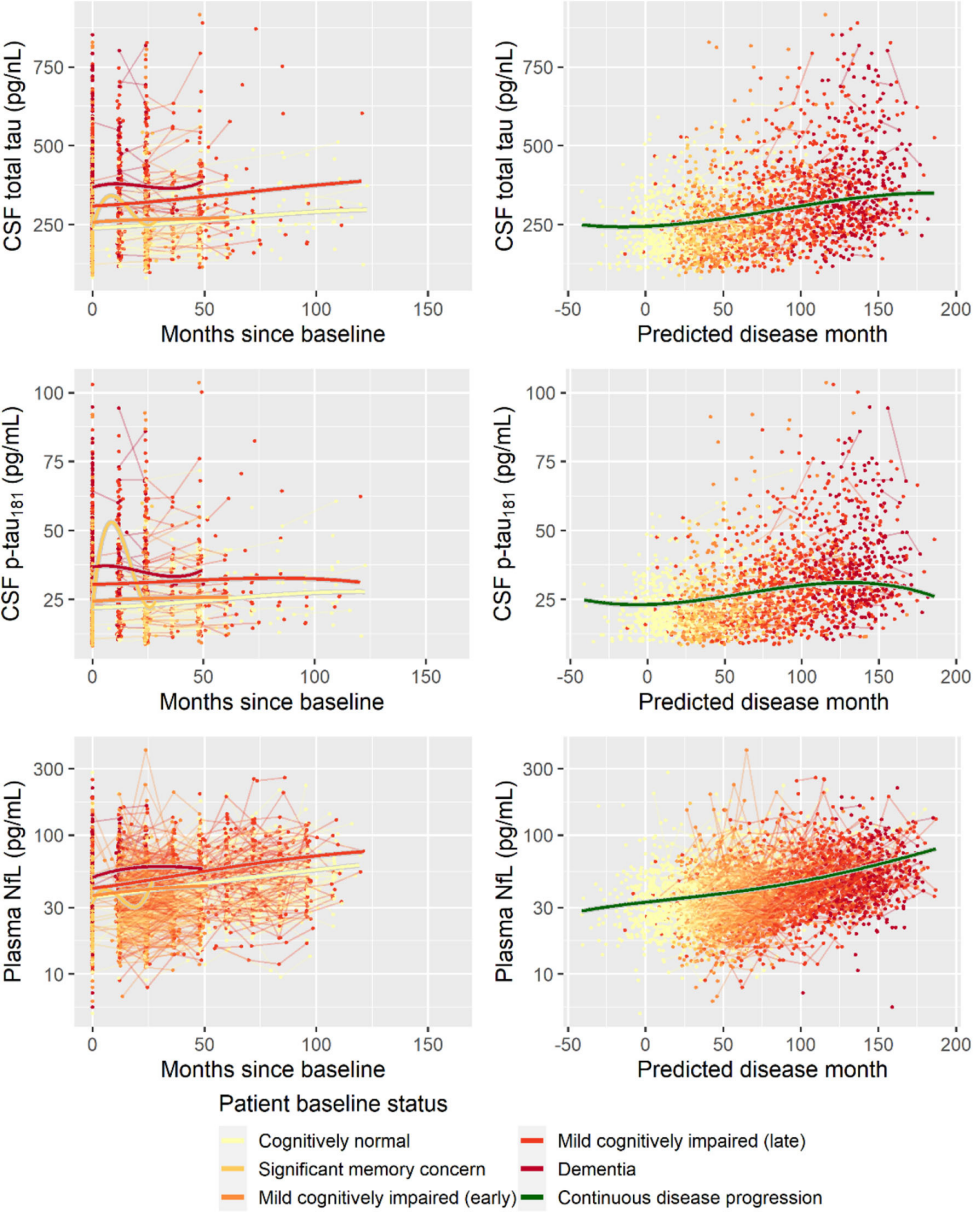
Data and estimated biomarker trajectories for total tau (CSF), *p*-tau_181_ (CSF), and NfL (plasma). Left column shows results when allowing different trajectories for the five baseline groups; right column shows the results when requiring a single trajectory over predicted disease time. Note that the oscillations for the significant memory concern group occur during time periods where no data were collected on the respective biomarkers.

Second, to validate that the predicted disease time also synchronized other independently captured aspects of the disease than cognition as measured by ADAS-Cog, we analyzed if the predicted continuous disease scale better captured patterns of variation in other clinical scales and biomarkers than separate modeling of the different baseline groups. We found that predicted disease time better described the patterns of variation compared to allowing separate patterns per baseline group in 7 of the 10 outcomes when measured by log likelihood (Table 1), even though the latter model had 16 degrees of freedom more than the former. When measured using AIC and BIC that both adjust for additional degrees of freedom to compare model quality, the predicted disease time model was better in 8 of the 10 cases for AIC and 10 of the 10 cases for BIC. Interestingly, the three biomarkers where group-wise modeling was better as measured by log likelihood were all measures related to amyloid burden (CSF Aβ_1−42_ and Aβ_1−42_/Aβ_1−40_ ratio, florbetapir PET). These biomarkers are known to have a bimodal distribution (Palmqvist et al., 2015) and are thus poorly modeled by a single trajectory. The estimated trajectories of the two types of models for cognitive scales are shown in Figure 3, imaging data trajectories are shown in Figure 4, and CSF and plasma biomarker trajectories are shown in Figures 5, 6. For some outcomes, the per-baseline group modeling approach had too many degrees of freedom for the significant memory concern and dementia groups. In these cases, the estimated mean trajectories oscillate during time periods where no data were collected. For the non-amyloid biomarkers, reducing the degrees of freedom for these group would have no bearing on the results in Table 1.

**Table 1:** Comparison of longitudinal modeling of clinical scales and biomarkers based on patient baseline group vs. continuous disease time.

**Outcome measure**	**Number of observations (number of patients)**	**One trajectory per baseline group (*****df*** **=** **24)**			**One trajectory across predicted disease time (*****df*** **=** **8)**		
		**−2·Log likelihood**	**AIC**	**BIC**	**−2·Log likelihood**	**AIC**	**BIC**
Clinical Dementia Rating Scale—sum of boxes	9,712 (2,142)	29,584.0	29,632.0	29,804.3	**29,062.3**	**29,078.3**	**29,135.8**
Functional Activities Questionnaire	9,715 (2,126)	51,328.2	51,376.2	51,548.5	**50,526.3**	**50,542.3**	**50,599.8**
FDG-PET (meta-ROI)	3,461 (1,454)	−7,185.3	−7,137.3	−6,989.7	**−7,594.8**	**−7,578.8**	**−7,529.6**
Hippocampal volume (MRI)	6,052 (1,675)	88,404.7	88,452.7	88,613.7	**88,372.1**	**88,388.1**	**88,441.7**
Florbetapir PET SUVr	2,568 (1,224)	**−3,466.3**	−3,418.3	−3,277.9	−3,430.2	**−3,414.2**	**−3,367.4**
Aβ_1−42_ (CSF)	2,342 (1,252)	**33,958.5**	**34,006.5**	34,144.7	34,011.1	34,027.1	**34,073.1**
Aβ_1−42_/Aβ_1−40_ (CSF)	1,425 (867)	**−5,838.2**	**−5,790.2**	−5,663.9	−5,816.5	−5,800.5	**−5,758.4**
Total tau (CSF)	2,334 (1,247)	26,441.1	26,489.1	26,627.2	**26,353.7**	**26,369.7**	**26,415.7**
p-tau_181_ (CSF)	2,330 (1,246)	15,849.8	15,897.8	16,035.9	**15,793.6**	**15,809.6**	**15,855.6**
NfL (plasma)	4,219 (1,576)	37,584.5	37,632.5	37,784.8	**37,517.8**	**37,533.8**	**37,584.6**

*Comparison in terms of −2·log likelihood, AIC and BIC (smaller is better for all measures). Bold numbers indicate the best-fitting model for a given measure*.

*df, degrees of freedom*.

### Age, Sex, Education, and Cognitive Decline

There were systematic differences in follow-up time, age at baseline, and length of education between male and female participants (Supplementary Table 1). Compared to female participants, male participants on average had 3.2 months' longer follow-up (Wilcoxon *p* = 0.0085), were on average 2.0 years older at baseline (Wilcoxon *p* < 0.0001), and had 0.89 years more education (Wilcoxon *p* < 0.0001). Age at baseline and years of education were not significantly correlated (Spearman ρ = −0.04, *p* = 0.0792).

**Figure 7:**
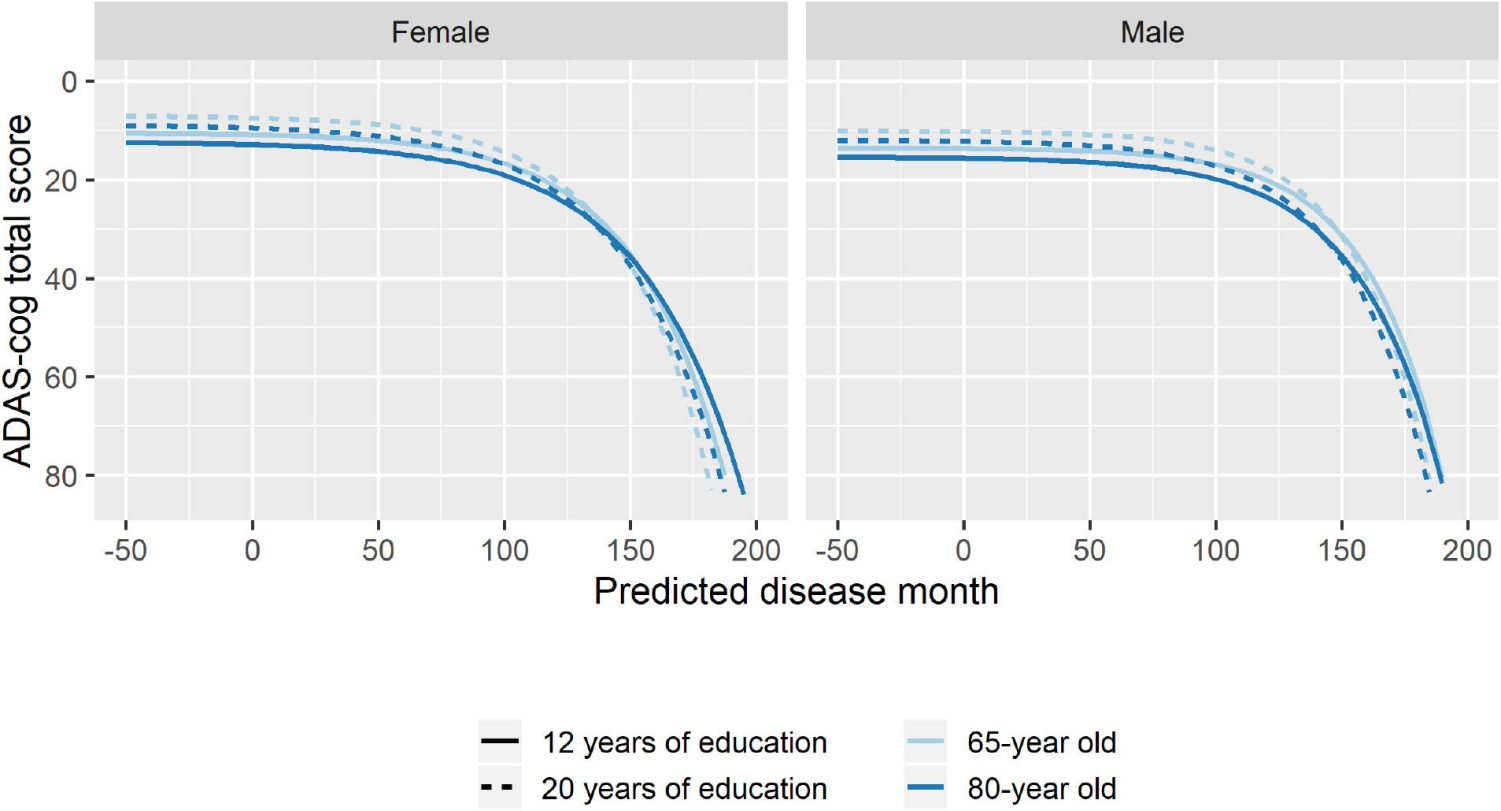
Estimated trajectories for different combination of patient age, sex, and length of education. The trajectories are aligned at predicted month 0 that corresponds to the average cognitive stage of cognitively normal individuals at baseline.

To explore whether age at baseline, sex, and length of education affected the pattern of cognitive decline, stepwise forward model selection was done to include these factors in the model. The best model included fixed covariate effects of age and sex on *g, s*, and *v*, and fixed covariate effects of years of education on *g* and *v*. While there were some substantial differences in marginal parameter estimates due to age, sex, and length of education (e.g., men are predicted to be 57 months later in disease compared to women in the same baseline groups; Supplementary Table 2), the estimates should not be interpreted in isolation because all parameters simultaneously affect the shape of the disease trajectory and may counteract each other. Figure 7 shows how age, sex, and education differences systematically affected the mean trajectories. From the figure, we see that male participants consistently scored lower on ADAS-Cog throughout the disease (3.1 points), but that they remained more stable in the initial 100 months where female participants had a more gradual decline. Lower age at baseline and longer education were both associated with higher cognitive scores, but also slightly increased rates of decline as evident in the stages of overt dementia (predicted disease time >120 months).

### Cholinesterase Inhibitors and Cognitive Decline

Using the search terms described in the Supplementary Material, we identified 1,347 individuals that were treated with ChEIs, which are approved for symptomatic treatment of AD. There were no restrictions to brand or dose used. Only 64 of the identified patients had records of initiation or discontinuation of treatment during the observation time (total of nine initiations and 60 discontinuations).

**Figure 8:**
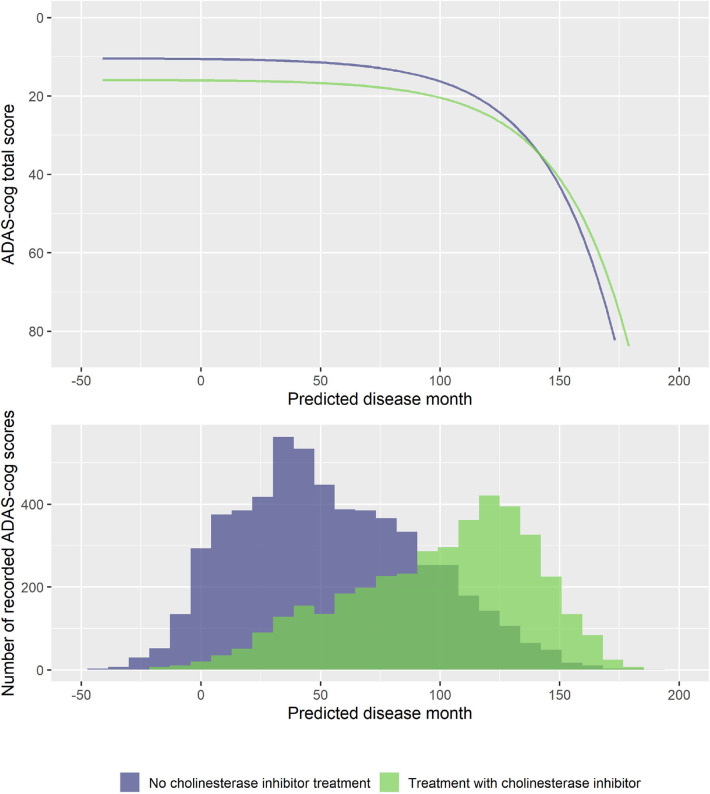
Estimated trajectories for patients with and without cholinesterase inhibitor treatment (top) and corresponding distribution of number of observed ADAS-Cog scores at corresponding predicted disease times. The trajectories are aligned at predicted month 0 that corresponds to the average cognitive stage of cognitively normal individuals that are not treated with cholinesterase inhibitors at baseline.

To explore if treatment with ChEIs affected the shape of the decline trajectories, stepwise forward model selection from the basic model was done to include ChEI treatment in the model. The best model included fixed effects of treatment on *s* and *v*, but not on rate of decline *g* (14 degrees of freedom, twice the negative log likelihood = −59,277.59, AIC = 59,305.59, BIC = 59,406.29). The model found that patients treated with ChEIs generally had worse level of cognition (effect on *v* was 5.50 ADAS-Cog points for treated individuals, *p* < 0.0001) and a delayed progression within baseline groups (effect on *s* was 7.53 months, *p* < 0.0001). The average trajectories and distribution of data across treatment are shown in Figure 8.

### Biomarkers for Disease Staging

The disease progression model relies on observing patients longitudinally and uses the temporal patterns of cognitive scores to predict the patient's status at baseline. This type of approach is needed for understanding the progression of disease and is valuable in retrospective cohort analyses. But the models presented thus far offer only little insight into the disease stage of a patient that has not been followed longitudinally, for example, a patient entering a clinical trial. In this setting, only the baseline classification of the patient, the cognitive score, and possibly other demographic data would be able to inform the stage of the patient. However, as shown in *Validation of the Basic Model*, several biomarkers have distinct temporal patterns over the course of predicted disease time. Biomarker data collected at baseline may thus enable a better assessment of the stage of an individual.

The following analyses were done on the 688 individuals who had complete biomarker data at baseline for the eight biomarkers considered in *Validation of the Basic Model*. These individuals had 3,301 visits with valid ADAS-Cog scores.

#### Training and Validation Data

Five hundred forty individuals (80%) were randomly selected for the training cohort, and the remaining 148 (20%) comprised the validation cohort.

#### Model Development

Using the BIC-based model selection procedure described previously, we searched for the best model among models that included adjustment for sex, baseline age, and education (on parameters *g, s, v*), as well as adjustment for the eight baseline biomarkers on disease stage (parameter *s*). The model selection was done on the training data. The best model included the biomarkers FDG-PET (meta-ROI), hippocampal volume (MRI), florbetapir PET SUVr, Aβ_1−42_/Aβ_1−40_ (CSF), and NfL (plasma) (22 degrees of freedom, −2·log likelihood = 15,182.34, AIC = 15,226.34, BIC = 15,355.45). The parameter estimates for the model are given in Supplementary Table 3.

#### Model Validation

**Figure 9:**
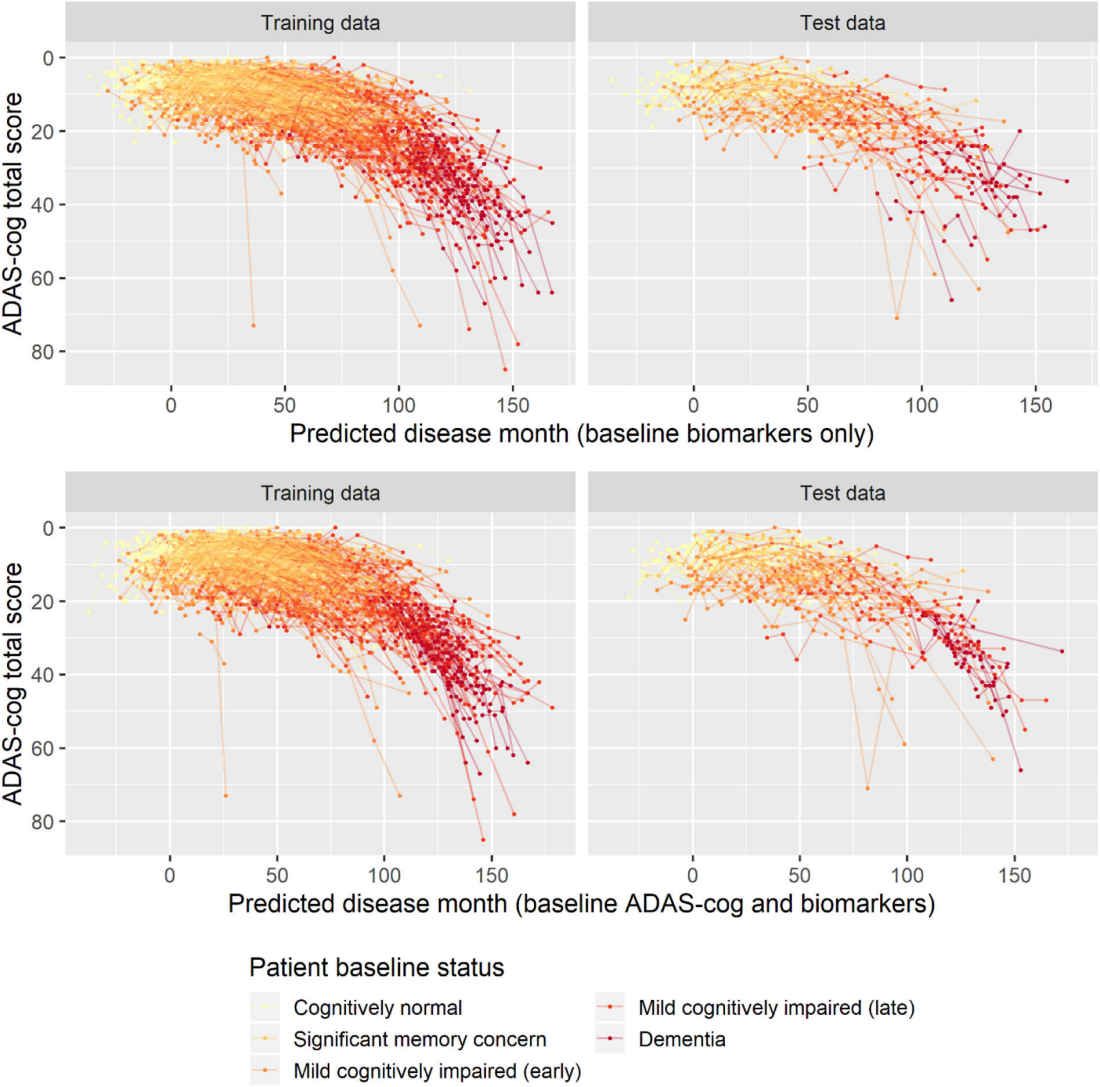
Predicted disease month for training and test datasets. Top row displays predicted disease-time alignment of observed ADAS-Cog total score trajectories based on baseline biomarker data and patient baseline status; bottom row displays predicted disease-time alignment of trajectories based on baseline ADAS-Cog total score, baseline biomarker data, and patient baseline status.

To validate the biomarker model, the model fitted on training data was used to predict disease stage in two different scenarios. In addition to patient status, the first used only baseline biomarker data, whereas the second also used baseline ADAS-Cog total score. Visual inspection of the longitudinal ADAS-Cog trajectories suggests that the baseline data do hold information that improves prediction of disease stage in the test data (Figure 9). To quantify this, the predictive accuracy of the biomarker model was compared to the basic model that did not include biomarker on the longitudinal ADAS-Cog total score trajectories (Table 2). Inclusion of biomarker data clearly reduced the mean squared error (MSE) and median absolute error (MAE) of predictions on both test and training data (MSE/MAE 65.1/4.21 vs. 100.0/4.98 on test data). Including the baseline ADAS-Cog total score improved the post-baseline predictive accuracy of the biomarker model further (MSE/MAE 55.1/3.48 for baseline biomarkers + ADAS-Cog model vs. 69.8/4.31 for biomarker model on test data).

**Table 2:** Predictive accuracies of predicted ADAS-Cog total score trajectories for the basic model and the biomarker model both with and without the baseline ADAS-Cog total score.

**Model and data**	**Mean squared error**		**Median absolute error**	
	**Training**	**Test**	**Training**	**Test**
Basic model (baseline status)	90.0	100.0 (110.6^a^)	5.48	4.98 (5.01^a^)
BM model (baseline status + BMs)	58.3	65.1 (69.8^a^)	4.19	4.21 (4.31^a^)
Basic model (baseline status + ADAS-Cog)	78.2^a^	102.7^a^	3.49^a^	3.70^a^
BM model (baseline status + ADAS-Cog + BMs)	53.5^a^	55.1^a^	3.29^a^	3.48^a^

*Predictions were censored to the interval [0, 85] to respect the range of the ADAS-Cog scores*.

*BM, biomarker*.

a *Baseline ADAS-Cog measurements excluded in computation of prediction errors*.

## Discussion

### Disease Progression Modeling

In this article, we presented a model for progression of dementia based on longitudinal cognitive assessments. Disease stages of individual patients were modeled using a latent variable approach. As opposed to conventional latent variable models, for example, those used in item response theory for modeling cognitive tests (Balsis et al., 2012; Embretson and Reise, 2013), the proposed model imposes explicit structures to ensure that the longitudinal modeling respects the known course of disease (e.g., that disease progression is an increasing function of elapsed time and that cognition on average declines with disease progression). By imposing these structures, the model provides a scaffold for understanding disease progression in pathological aging in terms of three continuous measures, *disease stage, rate of decline*, and *cognitive deviation from the mean*.

The proposed model aligned trajectories of cognitive decline. To demonstrate that this approach provided valid insights about other aspects of the disease, it was shown that predicted disease time was predictive of various measures of disease onset. Furthermore, the use of ADAS-Cog trajectories to map patients to a one-dimensional disease timeline was shown to consistently provide a better explanation of other clinical scales and biomarker trajectories than a conventional approach that grouped patients based on baseline symptoms.

The presented model was formulated for one-dimensional outcomes. This choice allowed formulation and implementation of the model in a non-linear mixed-effects modeling framework using maximum likelihood estimation. This in turn enabled modeling of covariate effects on different aspects of disease progression, sophisticated models for random variation in data, and the possibility of taking advantage of the large body of developed statistical methodology for mixed-effects modeling (Pinheiro and Bates, 2006). Because AD is multifaceted, and different measures are sensitive of disease stage and progression at different times during AD, progression models for multivariate outcomes can be more sensitive than univariate models. However, realistic specification of covariate effects, cross-covariance structures, and dependence between random effects for the different outcomes may be very difficult and require a large number of free parameters. While model classes and estimation procedures for similar longitudinal multivariate outcomes have been proposed in other fields (Olsen et al., 2018), existing multivariate models for AD progression generally rely on simple modeling of different sources of variation. Future work should address this gap in the current available methodology: while existing multivariate models can achieve high-quality staging of patients and outcomes with simple noise modeling by taking advantage of the aggregated information across many outcomes (Donohue et al., 2014; Jedynak et al., 2015), they can likely not take advantage of this aggregated information to address specific questions about whether a covariate affects a specific aspect of disease progression.

### Age, Sex, Education, and Cognitive Decline

The effect of demographic and socioeconomic factors on disease risk and manifestation in AD has been the subject of much study. In this work, we focused on the combined effects of age, sex, and length of education.

When considered individually, these factors have been observed to result in differences in disease progression. While age is typically considered the major risk factor for developing AD, higher age at AD onset has been observed to be associated with a slower rate of cognitive decline (Gardner et al., 2013; Stanley et al., 2019). Similarly, female sex has been identified as a major risk factor, with almost two-thirds of AD cases being women (Alzheimer's Association., 2018). While this difference has been known for a long time, it has only become apparent more recently that there are sex differences in symptomatology, rate of decline, and possibly in neural anatomy (Ferretti et al., 2018; Oveisgharan et al., 2018). The effects of cognitive reserve on age-related cognitive decline have been the subject of much study (Tucker and Stern, 2011). Cognitive reserve is often studied using educational attainment as an operational proxy for cognitive reserve. It has consistently been found that higher education is associated with increased rate of cognitive decline in incident AD (Teri et al., 1995; Rasmusson et al., 1996; Wilson et al., 2004; Andel et al., 2006; Scarmeas et al., 2006; Musicco et al., 2009; Thomas et al., 2016), with several of these studies also reporting that education is associated with higher baseline cognition.

Differences in cognitive decline are often studied by comparing slopes in statistical models that assume that cognitive decline follows a linear pattern. The argumentation and interpretation around the cognitive reserve model are somewhat more sophisticated, but still largely centered on an assumption of a linear rate of decline (e.g., illustrated in Figure 1 in Stern, 2012). The prevailing hypothesis within the field of cognitive reserve research is that, compared to individuals with low cognitive reserve, individuals with high cognitive reserve have higher pre-disease cognitive scores and that their brains tolerate a higher load of neuropathology before cognitive decline is seen. At a sufficiently high level of neuropathology, cognitive ability reaches its floor for all participants. If the timescale of neuropathological buildup is similar across individuals, this suggests that individuals with high cognitive reserve will have to decline a wider range of cognitive scores in a shorter time, thus leading to an accelerated rate of decline (Stern, 2012).

The analyses in the present article clearly illustrate that rate of cognitive decline as measured on ADAS-Cog is not constant but increases over the course of AD. Thus, findings of an increased rate of decline in a certain group of patients using slope models could either be because the group of patients has accelerated decline, because they are at a later disease stage, or a combination. The proposed disease progression model seeks to align cognitive trajectories on a disease timeline, and thus it allows one to separate the hypothesized mechanisms of cognitive decline. The best model that adjusted for effects of age at baseline, sex, and length of education on, respectively, disease stage, rate of decline, and cognitive deviation found that all three factors affected all three disease measures except for disease stage, which was not affected by length of education.

When considering the combination of effects (Figure 7), the results suggested that higher age at baseline was associated with lower cognition throughout disease time and a slightly reduced rate of decline. Women tended to have not only better pre-disease cognition but also an accelerated decline. Finally, longer education was associated with slightly faster rate of decline and a systematically better cognition throughout the disease.

While these findings are largely consistent with previous findings, they also illustrate that previous results that do not take the long-term disease trajectories into account may be systematically biased. In particular, the fact that highly educated patients tend to have above-mean cognition throughout the early stages of disease means that they will meet cognitive cutoffs used for inclusion criteria in clinical studies longer into their disease than patients with less education. Because of the accelerated cognitive decline in the later stages of disease, these patients will have a much faster rate of decline when using conventional slope models, but this difference will primarily be due to their later disease stage.

### Symptomatic Medications for Alzheimer Disease and Cognitive Decline

Cholinesterase inhibitors have consistently shown a symptomatic benefit in mild to severe dementia due to AD in randomized, double-blind, placebo-controlled trials (Birks, 2006). It has, however, been questioned whether long-term treatment with ChEIs could be harmful (Schneider, 2012). A recent meta-analysis found that AD patients treated with symptomatic treatments had a faster rate of cognitive decline (Kennedy et al., 2018). This could be interpreted as a harmful side effect, but because the included studies were not randomized with respect to symptomatic treatments, such causal link cannot be made. An alternative explanation is simply that ChEIs work—that patients who are being treated at study inclusion have a cognitive benefit that, similarly to higher levels of education, means that they meet inclusion criteria for clinical studies further into their disease. The optimal disease progression model identified in the model search did not include effects of ChEI treatment on rate of decline. Instead, the results of this model showed that patients treated generally had lower cognition compared to untreated patients (which points to confounding by indication; patients are prescribed ChEIs because of their cognitive impairment) and that their progression was slightly delayed.

### Biomarker-Based Disease Staging

The final application of the model examined how a patient's biomarker profile at study entry could be used to predict his/her disease stage. Based on training data used for model development, a set of five biomarkers were included in the model. Biomarker profiles considerably improved prediction of future ADAS-Cog trajectories in the unseen validation dataset, and inclusion of baseline ADAS-Cog score further improved the prediction. Among the biomarkers, FDG-PET explained most variation followed by CSF Aβ_1−42_/Aβ_1−40_ and florbetapir SUVr. Hippocampal volume and plasma NfL explained the least.

This modeling of baseline biomarkers for patients in the earliest stages of disease takes advantage of the long-term follow-up that is unique to ADNI. The modeling essentially relies on hindsight because the patients' disease stage can only be predicted with high reliability once a systematic pattern of cognitive decline has been observed. By using these patterns, the model identified how combinations of biomarkers could be used to predict disease stage. The results of the model suggest that biomarker profiles at a single time point may be used to predict the disease stage of an individual even in the preclinical phases of disease where no clinically detectable cognitive impairment is present.

With further validation, these results can be used to define a space of permissible biomarker profiles to use as inclusion criteria in clinical trials. Such biomarker-based synchronization of patient's disease stage would enable testing a drug in a more homogeneous population. This would in turn greatly increase the power of clinical trials in AD where it is common to see extreme levels of variability in patient trajectories (Cummings et al., 2018; Ballard et al., 2019).

## Data Availability Statement

ADNI data can be accessed after accepting its data use agreement and submitting an online application for access. For more information, please see the ADNI website http://adni.loni.usc.edu/.

## Author Contributions

LR developed the disease progression model, analyzed the data, and wrote the paper.

## Conflict of Interest

LR was employed by company H. Lundbeck A/S.
