# Supplementary material for: Statistical Disease Progression Modeling in Alzheimer Disease
Source: Front Big Data. 2020 Aug 12;3:24. doi: 10.3389/fdata.2020.00024 (PMC7931952; doi:10.3389/fdata.2020.00024)
Supplement: Supplementary file 1 [file Data_Sheet_1.docx]

# Supplementary material for Statistical disease progression modeling in Alzheimer’s disease

Lars Lau Raket

**Cholinesterase inhibitor treatment data extraction and processing**

Treatment status with cholinesterase inhibitors was extracted from data on key background medications and the concurrent medications log. The latter was searched for the following common names and misspellings *‘aricept’, ‘aricpet’, ‘donepezil’, ‘exelon’, ‘galantamine’, ‘razadyne’, ‘remenyl’, ‘reminyl’, ‘rivastigmine’*.

Missing month and day in dates were interpolated as follows: missing day was set to the 1^st^ day of the month. Missing month was set to July.

***Supplementary Table 1*** *Sex differences.*

|  | Female | Male | p-value* |
| --- | --- | --- | --- |
| *Participants, n* | 1,002 | 1,140 | — |
| *ADAS-cog scores, n* | 4,364 | 5,466 | — |
| *Follow-up time (months), median [IQR]* | 24.3 [5.5, 59.5] | 25.4 [11.9, 60.6] | 0.0085 |
| *Age at baseline (years), median [IQR]* | 72.0 [57.4, 77.5] | 74.3 [69.5, 79.3] | < 0.0001 |
| *Length of education (years), median [IQR]* | 15 [14, 18] | 16 [15, 18] | < 0.0001 |

*p-values computed using Wilcoxon rank sum tests

IQR: Interquartile range

***Supplementary Table 2*** *Parameter estimates in model adjusting for age, sex and education. p-values are computed using likelihood ratio tests.*

| Parameter | Estimate | p-value |
| --- | --- | --- |
| *l (intercept)* | 0.01337 | — |
| *s (significant memory concern)* | 35.11 | 0.0106 |
| *s (early MCI)* | 44.58 | < 0.0001 |
| *s (late MCI)* | 87.73 | < 0.0001 |
| *s (dementia)* | 136.1 | < 0.0001 |
| *s (age at baseline)* | 1.878 | < 0.0001 |
| *s (male)* | -57.15 | < 0.0001 |
| *g (intercept)* | 3.169 | — |
| *g (age at baseline)* | 0.007061 | < 0.0001 |
| *g (male)* | -0.1895 | < 0.0001 |
| *g (years of education)* | -0.003337 | 0.0011 |
| *v (intercept)* | 7.623 | — |
| *v (age at baseline)* | 0.1234 | < 0.0001 |
| *v (male)* | 3.113 | < 0.0001 |
| *v (years of education)* | -0.4285 | < 0.0001 |

***Supplementary Table 3*** *Parameter estimates in model adjusting for age, sex, education and biomarkers. p-values are computed using likelihood ratio tests.*

| Parameter | Estimate | p-value |
| --- | --- | --- |
| *l (intercept)* | 17.92 | — |
| *s (significant memory concern)* | 18.20 | 0.0663 |
| *s (early MCI)* | 13.14 | < 0.0001 |
| *s (late MCI)* | 40.00 | < 0.0001 |
| *s (dementia)* | 64.12 | < 0.0001 |
| *s (FDG-PET)* | -73.52 | < 0.0001 |
| *s (hippocampal volume)* | -0.005980 | < 0.0001 |
| *s (Florbetapir SUVr)* | 34.57 | < 0.0001 |
| *s (CSF Aβ1-42/Aβ1-40)* | -137.1 | < 0.0001 |
| *s (plasma NfL)* | 0.2579 | < 0.0001 |
| *g (intercept)* | 3.280 | — |
| *g (age at baseline)* | 0.01276 | < 0.0001 |
| *g (male)* | -0.3764 | < 0.0001 |
| *g (years of education)* | -0.03897 | 0.0008 |
| *v (intercept)* | 11.71 | — |
| *v (male)* | 4.026 | < 0.0001 |
| *v (years of education)* | -0.2133 | 0.217 |
